# Partial transformation from non-small cell lung cancer to small cell lung cancer: a case report and literatures review

**DOI:** 10.3389/fonc.2025.1441182

**Published:** 2025-03-11

**Authors:** Yicong Lu, Danruo Fang, Jiangying Guo, Huaqiong Huang

**Affiliations:** Key Laboratory of Respiratory Disease of Zhejiang Province, Department of Respiratory and Critical Care Medicine, Second Affiliated Hospital of Zhejiang University School of Medicine, Hangzhou, Zhejiang, China

**Keywords:** SCLC, NSCLC, transformation, EGFR mutation, treatment

## Abstract

A fraction of lung adenocarcinoma patients with gene mutations who receive targeted therapy would experience acquired resistance and undergo small cell lung cancer (SCLC) transformation. The mechanisms behind the transformation of tumor pathological types and the treatment strategies are not fully clear. There have been case reports of the transformation from adenocarcinoma to SCLC, but the partial transformation from adenocarcinoma to SCLC has not been reported. We reported a case of a patient with partial transformation from lung adenocarcinoma to SCLC for the first time. The patient was diagnosed as lung adenocarcinoma with epidermal growth factor receptor (EGFR) 19 exon mutation and Tumor protein p53 (TP53) mutation. She received Epithelial growth factor receptor tyrosine kinase inhibitors (EGFR-TKIs) treatment. However, the tumor progression occurred and the lung aspiration pathology revealed a transformation from non-small cell lung cancer (NSCLC) to SCLC. The treatment regimen was changed to cisplatin and etoposide (EP) chemotherapy, resulting in a 2-month PFS. It was worth mentioning that adenocarcinoma cells were found in the patient’s emerging pericardial effusion, suggesting the co-existence of both adenocarcinoma and SCLC components. This is the first report of partial transformation from NSCLC to SCLC in the context of definitive pathology. It highlights that when no more pathological biopsy is feasible, we should be alert to the partial transformation and adopt the appropriate treatment.

## Introduction

Common mutation types in lung adenocarcinoma include epidermal growth factor receptor (EGFR), anaplastic lymphoma kinase (ALK) rearranged, and C-ros oncogene 1-receptor tyrosine kinase (ROS1) fusion (1). EGFR is a common mutation type, with somatic EGFR mutations detected in 30-40% of Asian NSCLC patients and approximately 10-20% of European or American NSCLC patients (2). Targeted therapy is possible for gene mutations (1). Although molecular targeted therapy can prolong the survival of patients and bring significant benefits, drug resistance inevitably occurs during treatment. The transformation from NSCLC to SCLC is one of the drug resistance mechanisms. It has been reported that 4% - 14% of NSCLC patients with EGFR mutations experience histological transformation to SCLC (3). However, the inherent mechanism and specific tumor marker changes are still in the hypothetical stage which have been the research hots and difficulties of clinical research. The treatment plans after the transformation have also been insufficiently studied.

There are no reports of histological partial transformation to SCLC in EGFR-driven lung adenocarcinoma. Therefore, we present a case of partial transformation from lung adenocarcinoma to SCLC after targeted therapy. The purpose of this study is to provide more clinical references for predictive factors, relevant mechanisms, and treatment strategies.

## Case report

**Figure 1:**
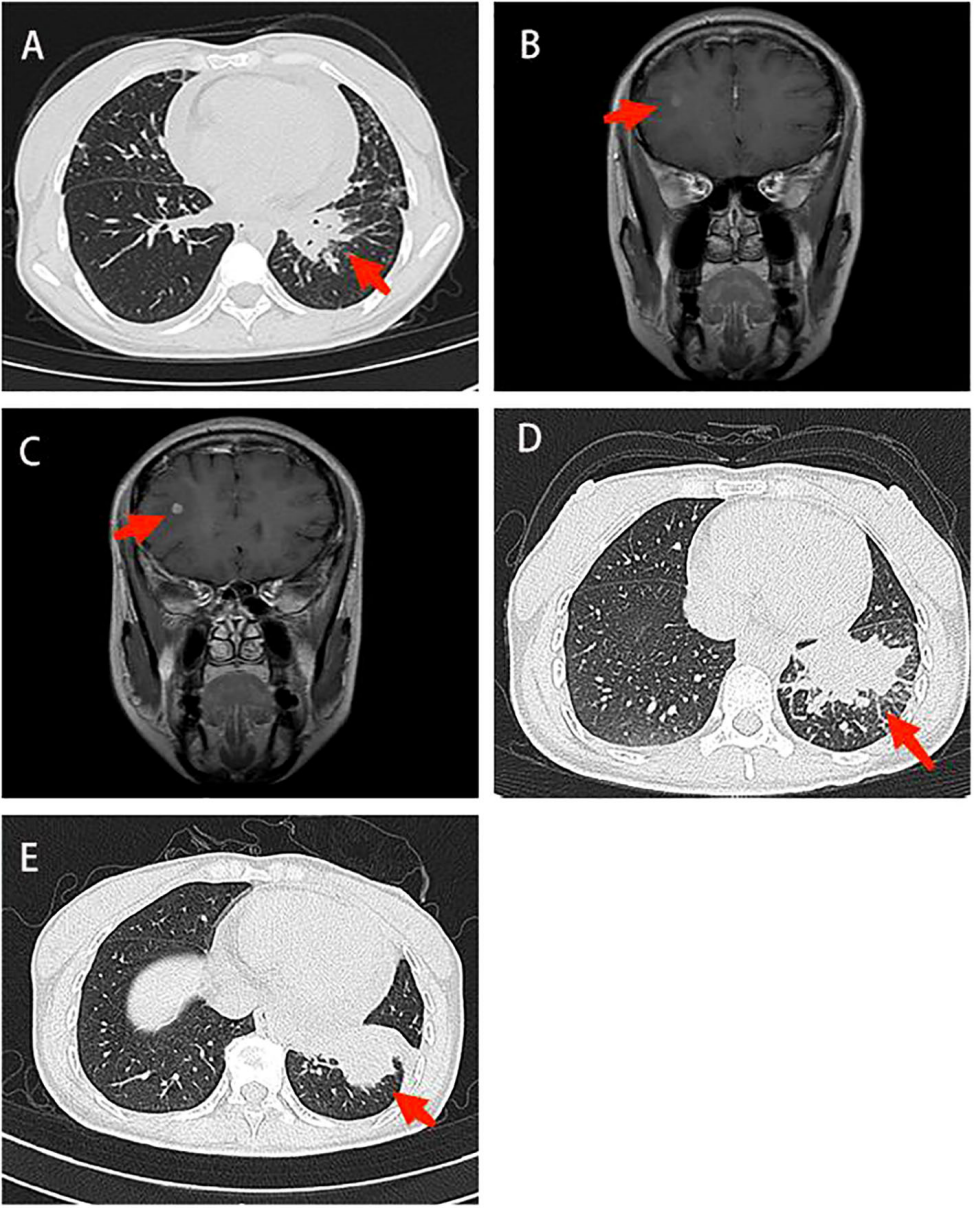
Imaging in diagnosis and treatment. **(A)** Chest CT: There was a mass shadow in the basal segment of the left lower lobe **(B)** Contrast-enhanced brain MRI: The right frontal lobe and basal ganglia were enhanced, and the possibility of metastasis was considered **(C)** Contrast-enhanced brain MRI: The enhancement foci in the right frontal lobe was enlarged compared to the anterior radiograph (2022–11–09). **(D)** Contrast-enhanced chest CT: The lung cancer in the basal segment of the left lower lobe, significantly enlarged compared to the anterior radiograph (2023–06–07). **(E)** Chest CT: Lung cancer in the left lower lobe associated with obstructive inflammation, shrank compared to the anterior radiograph (2023–07–14). CT, computed tomography; MRI, magnetic resonance imaging.

**Figure 2:**
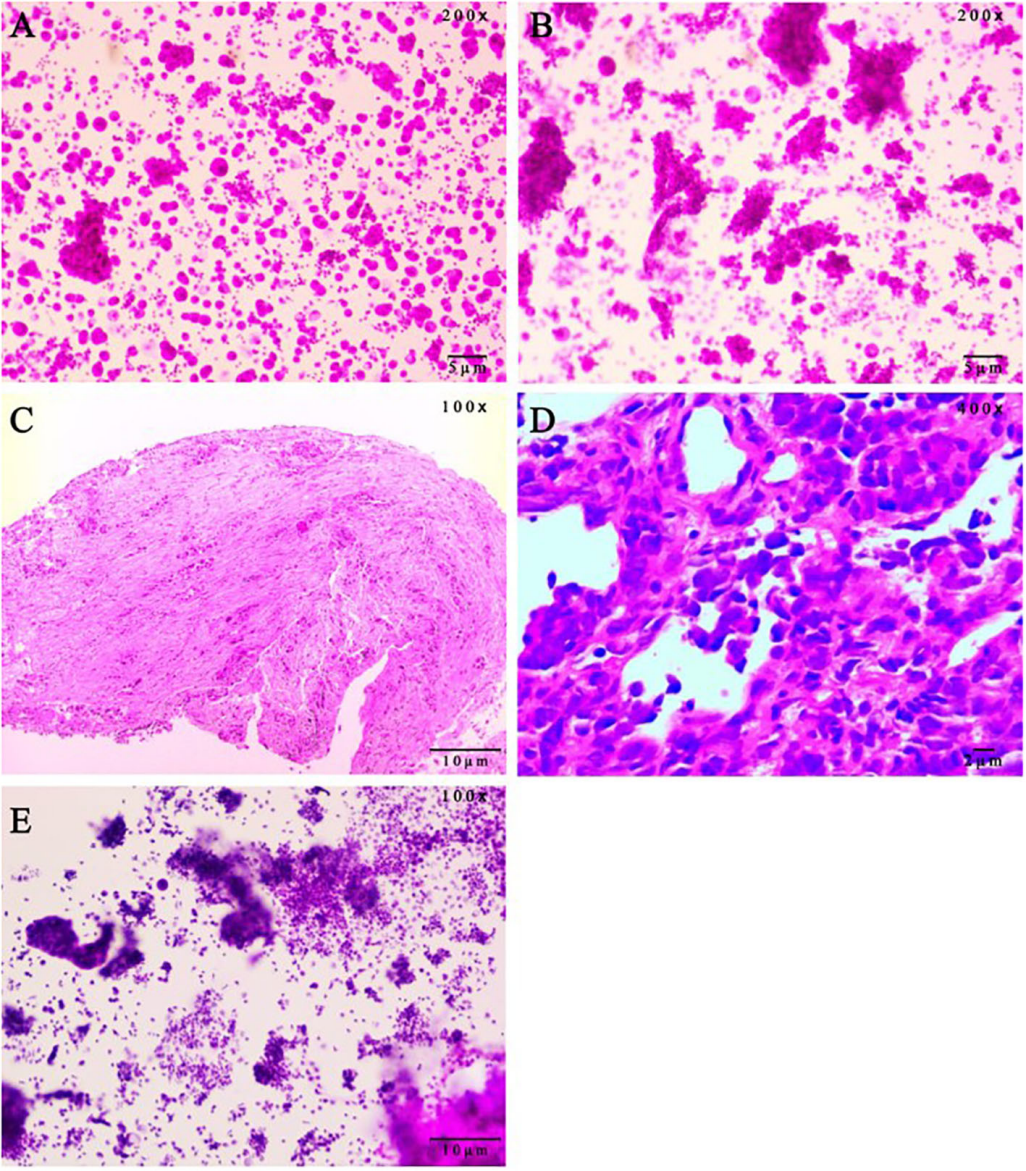
Pathological evaluation on patient samples. **(A)** Malignant tumor cells were found in pericardial effusion smear, immunohistochemistry (Calretinin mesothelium +, NapsinA +, CEA(Mono) +, TTF-1 +, CK7 +, WT1 mesothelium +, P63 -, ALK-Lung +/-, ALK-Lung-NC -, EMA +, ER -, PR -, CDX2 -, PAX-8 -, GATA-3 -) showed lung adenocarcinoma. (hematoxylin and eosin staining, 200x magnification). **(B)** Malignant tumor cells were found in hydrothorax smear. (hematoxylin and eosin staining, 200x magnification). **(C)** Bronchoscopic pathology showed adenocarcinoma in the basal segment of the left lower lobe. Immunohistochemistry results were ALK-Lung -, ALK-Lung-NC -. (hematoxylin and eosin staining, 100x magnification). **(D)** Lung aspiration Pathology showed poorly differentiated lung cancer, and immunohistochemical staining (CK(AE1/AE3) +, TTF1(8G7G3/1) -, P40 -, CK5/6 -, Syn +, CgA +, CD56 +, INSM1 +, CK20 -, Ki-67 90%) was consistent with SCLC. (hematoxylin and eosin staining, 400x magnification). **(E)** Adenocarcinoma cells were found in pericardial effusion smear. The scale bars were indicated in the bottom right corner of the pictures. SCLC, small-cell lung cancer. (hematoxylin and eosin staining, 100x magnification).

On September 4, 2020, a 33-year-old Chinese woman visited our department due to chest tightness and shortness of breath after activity. She had no profession. She never smoked or drank, and there was no special family or personal history. However, elevated carcinoembryonic antigen and carbohydrate antigen were detected, and chest high-resolution computed tomography (CT) suggested a mass shadow in the basal segment of the left lower lobe accompanied by scattered small nodules in the left lung (**Figure 1A**). Adenocarcinoma cells were found on both in pericardial and pleural effusion smears (**Figures 2A, B**). The pathological result of bronchoscopy was adenocarcinoma (basal segment of the left lower lobe), and metastatic adenocarcinoma was seen in the 4R group lymph node puncture (**Figure 2C**). The results of gene detection were EGFR exon 19 mutation and Tumor protein p53 (TP53) mutation. The patient was finally diagnosed with”left lung adenocarcinoma (T3N3M1a IVA stage, EGFR exon 19 mutation and TP53 mutation)”. After excluding the contraindications of chemotherapy, Icotinib combined with pemetrexed and nedaplatin were administered as first-line therapy. On February 1, 2021, the curative effect was evaluated as stable disease (SD). Icotinib and pemetrexed were adopted to maintain chemotherapy for 13 times, and then icotinib was used alone for targeted therapy.

On April 14, 2022, Contrast-enhanced brain magnetic resonance imaging (MRI) revealed an enhanced lesion in the right frontal lobe and basal ganglia (**Figure 1B**). Combined with the clinical presentation, the possibility of metastatic tumors was considered. Therefore, the physician changed the treatment from icotinib to osimertinib as second-line therapy. At the same time, the patient received 6MV X-ray D95 pGTV 32.5Gy/5F SBRT radiotherapy to the metastatic tumor area in the right frontal lobe and basal ganglia for a total of 5 times from May 30, 2022. On January 6, 2023, the contrast-enhanced brain MRI showed an enlarged enhanced lesion in the right frontal lobe compared with the previous image (2022–11–09) (**Figure 1C**). Therefore, on January 12, 2023, on the basis of Osimertinib, bevacizumab was given intravenously 5 times.

**Figure 3:**
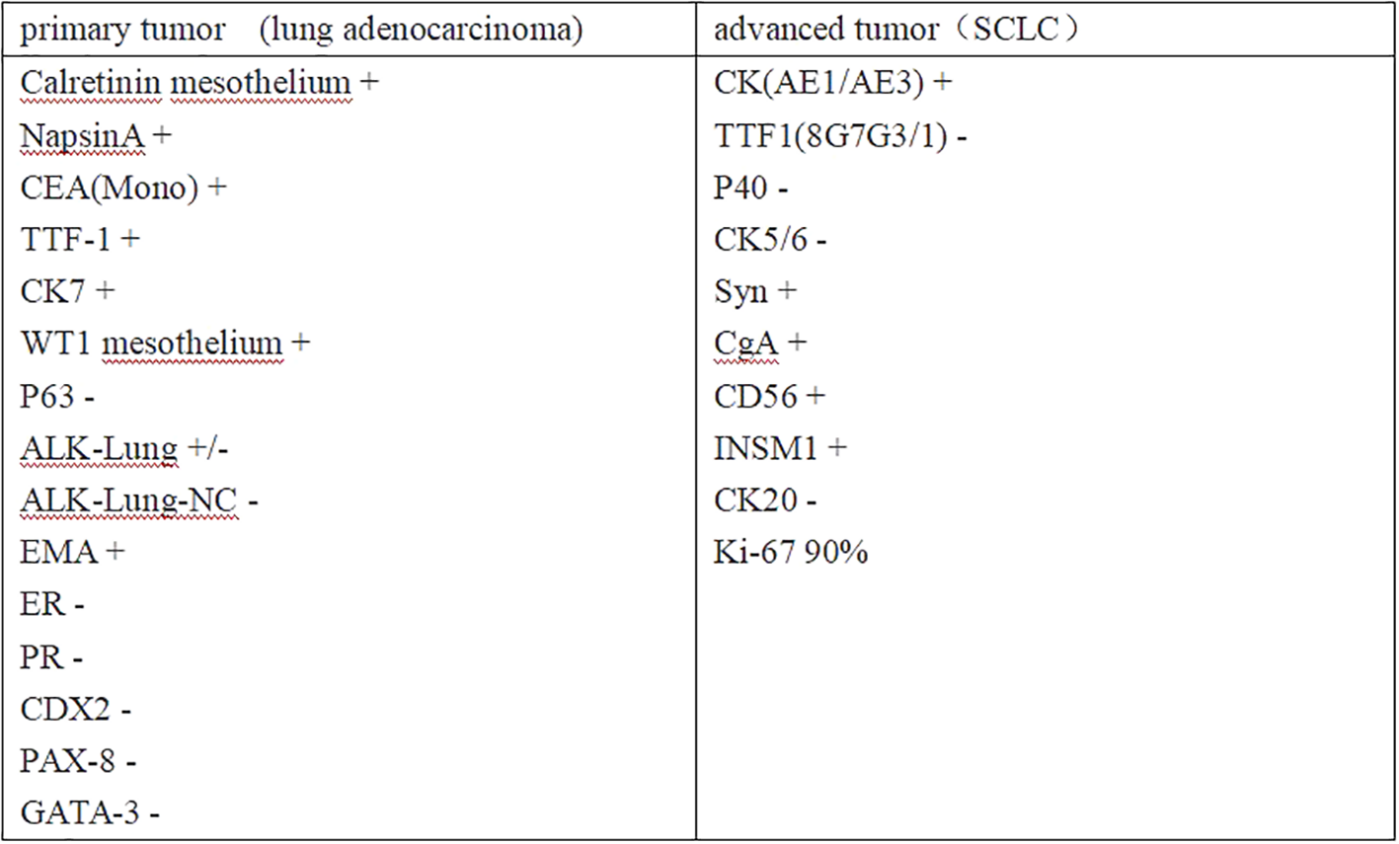
Panel plot of the main IHC results of primary and advanced tumor components.

On July 14, 2023, Contrast-enhanced chest CT suggested lung cancer in the basal segment of the left lower lobe, and the cancer foci was enlarged compared with the previous image (2023–06–07) (**Figure 1D**). At the same time, neuron-specific enolase (NSE) and squamous carcinoma-associated antigen both elevated by 93.9 ng/mL and 23.5 ng/mL, respectively. CT-guided lung puncture biopsy was performed on July 14, 2023, and the pathology combined with immunohistochemical staining report were consistent with SCLC (**Figure 2D**). A panel figure with the main immunohistochemical findings of the primary tumor and the progressive tumor was in **Figure 3**. At the same time, the gene detection was EFGR (+). The patient’s tumor pathology had changed. Thus, the diagnosis was as follows: 1. Malignant tumor of the left lung (rT3N3Mx SCLC, EGFR mutation-positive); 2. Left lung adenocarcinoma (T3N3M1a IVA stage, with metastasis to the pleura, pericardium, and brain, EGFR exon 19 mutation, TP53 mutation). The treatment was changed to cisplatin and etoposide (EP) chemotherapy regime from July 26, 2023.

**Figure 4:**
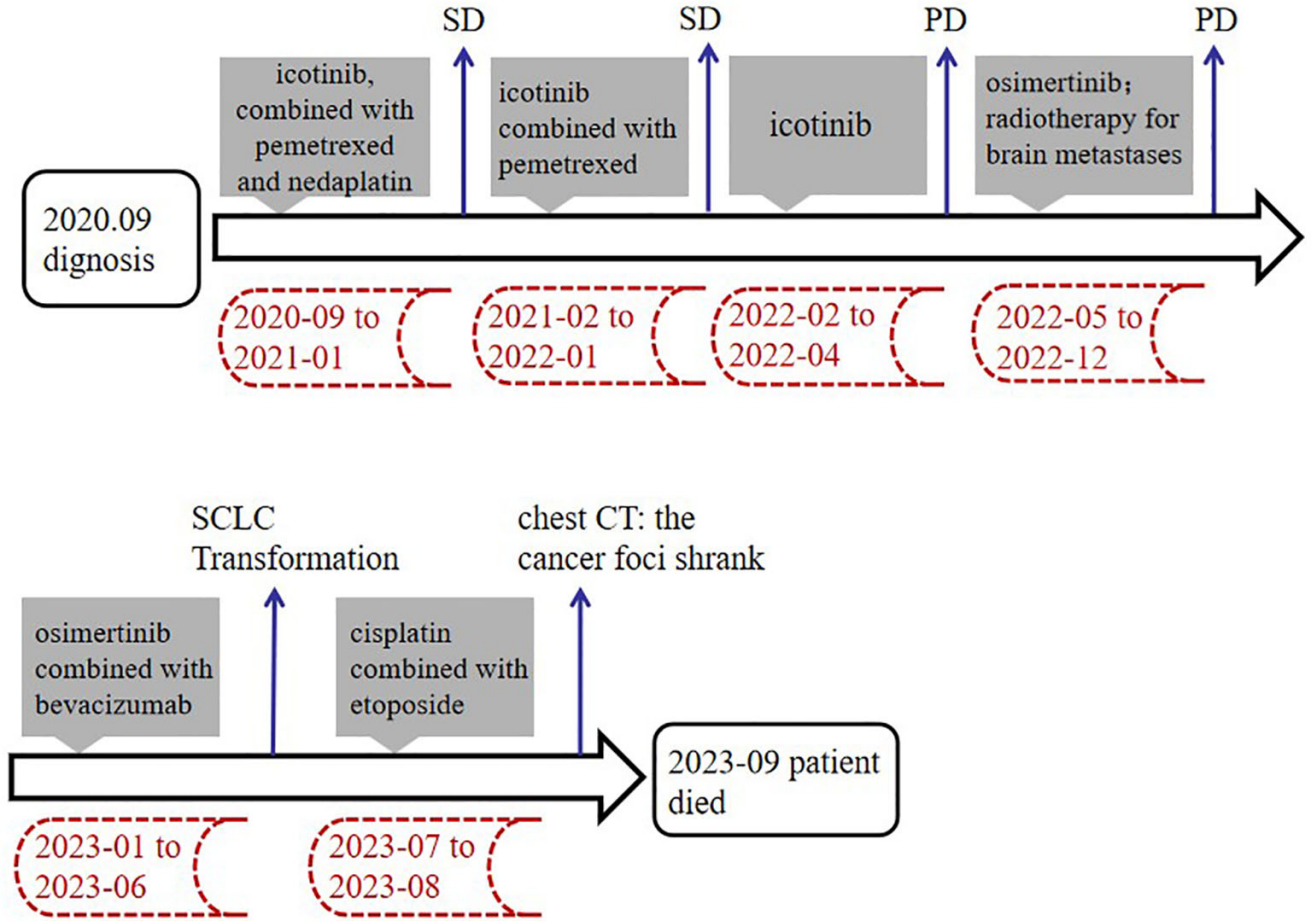
Summary of treatment timeline. SD, stable disease; PD, progressive disease.

On September 1, 2023, the patient began to experience chest tightness and chest pain discomfort without any obvious inducement and the symptoms were more severe after activity. A color ultrasound examination suggested a large amount of pericardial effusion (with mild filling signs). Emergency pericardial puncture and tube placement were performed. On September 6, 2023, the pathology report showed adenocarcinoma cells in the pericardial effusion smear (**Figure 2E**). On September 7, 2023, The chest high-resolution CT scan suggested lung cancer in the lower lobe of the left lung with obstructive inflammation, the cancer foci was smaller than in the previous image (2023–07–14) (**Figure 1E**). On the morning of September 7, 2023, the patient began to experience mental confusion and sleepiness, which later turned into irritability and restlessness. An emergency brain CT scan showed no significant acute symptoms. After a consultation with a neurologist, lung cancer and brain metastases were considered. As the prognosis was poor, the patient chose to be discharged automatically and transferred to a local hospital for further treatment. The patient eventually died of multiple tumor metastases and severe complications. The treatment timeline of the patient was presented in **Figure 4**.

## Discussion

We report a case of partial transformation to SCLC in EGFR-driven lung adenocarcinoma for the first time. After the transformation, adenocarcinoma cells were observed in the pericardial effusion smear, indicating the coexistence of the original cancer type with the converted cancer type. Since the patient died of multiple tumor metastases and severe complications after discharge, we were unable to perform multi-site biopsies of the primary cancer foci to confirm the presence of two pathological types in the primary tumor.

Currently, there are two hypotheses about the mechanism of NSCLC transformation to SCLC: One hypothesis is the lineage plasticity hypothesis: Type II alveolar cells have the ability to differentiate into adenocarcinoma and SCLC. Under the selective pressure of TKI treatment, lung adenocarcinoma with EGFR mutations caused by these type II alveolar cells may be re-differentiated into SCLC (4, 5). The evidence comes from studies that have shown consistent gene phenotypes in the original NSCLC lesions and the transformed SCLC (6–8). EFGR (+) was detected at the same time, while EGFR mutation was very rare in SCLC (3, 9). Another hypothesis is the clonal selection hypothesis: SCLC and NSCLC coexist in the primary tumor, and after first-line treatment, the number of NSCLC cells decreases, while the SCLC component becomes dominant, presenting as the so-called SCLC transformation (4).

Although the whole-exome sequencing demonstrated that the transformed SCLC shares a common clonal origin with the original adenocarcinoma (shared TP53/RB1 mutations) (10). However, the median time for SCLC transformation is actually 19 months (11). In the clonal selection hypothesis, it is difficult to explain how the SCLC progress slowly while the patients respond well to EGFR-TKI drugs for such a long treatment period. What’ more, the coexistence ofadenocarcinoma and SCLC components in this case challenges the clonal selection model, which predicts mutual exclusivity. Instead, the retention of EGFR mutations in transformed SCLC and the universal RB1/TP53 inactivation strongly favor lineage plasticity. This is further supported by the prolonged TKI response (13 cycles), inconsistent with rapid clonal expansion (8, 12, 13). And the whole-exome sequencing demonstrated that the transformed SCLC shares a common clonal origin with the original adenocarcinoma (shared TP53/RB1 mutations). Therefore, the homologous transformation mechanism may be more credible. Future studies should integrate multi-region sequencing to resolve spatial heterogeneity and validate plasticity mechanisms.

In our case report, the patient initially had lung adenocarcinoma with EGFR and TP53 mutations. After treatment with EGFR-TKIs, there was tumor progression, and a biopsy showed transformation to SCLC. However, adenocarcinoma cells were still found in the pericardial effusion later on. This suggests that both cancer types were present simultaneously, not completely replaced. So, the coexistence might represent different clones evolving under treatment pressure. The original adenocarcinoma might have partially transformed into SCLC, but residual adenocarcinoma cells persisted, possibly in different anatomical sites. The temporal progression aligns with the hypothesis that under EGFR-TKI selective pressure, a subclone of the original adenocarcinoma—harboring additional genetic alterations (e.g., RB1/TP53 loss)—acquired neuroendocrine features and proliferated as SCLC. However, residual adenocarcinoma cells, potentially located in protected niches (e.g., pericardium), evaded treatment and continued to grow. This coexistence implies intratumoral heterogeneity, where distinct clones evolve independently.

Some studies have interpreted the transformation of SCLC at the molecular level and predicted the characteristics related to the transformation. The lack of retinoblastoma protein (Rb) and protein 53 (P53) expression is considered to be one of the molecular mechanisms of SCLC transformation (3, 10, 14). RB1 is a tumor suppressor gene involved in cell cycle control, and its loss is common in SCLC. TP53 is another tumor suppressor, and its mutation leads to genomic instability. RB1 loss disrupts the G1/S cell cycle checkpoint, enabling uncontrolled proliferation and lineage plasticity. In lung adenocarcinoma, RB1 deletion or mutation is rare but becomes prevalent in transformed SCLC, facilitating neuroendocrine differentiation. TP53 mutations impair DNA repair and apoptotic responses, promoting genomic instability. This accelerates the accumulation of additional driver mutations (e.g., RB1 loss) under TKI pressure (15–17). Relevant studies have shown that when EGFR/Retinoblastoma 1 (RB1)/Tumor protein p53 (TP53) were all mutated, the relative risk of SCLC transformation increased by 43 times. RB1 and TP53 inactivation have been reported as markers of transformation from lung adenocarcinoma with EGFR mutation, ROS1 fusion, and ALK rearrangement to SCLC (12, 18–21). The patient’s TP53 mutation may have primed her tumor for this transformation, while RB1 loss (though not explicitly tested here) is strongly implicated in neuroendocrine reprogramming. These mutations collectively enable a “cell fate switch” from adenocarcinoma to SCLC. Future work should emphasize routine RB1/TP53 testing in EGFR-mutant adenocarcinomas to identify high-risk patients and guide early intervention. Rapid elevation of serum neuron specific enolase (NSE), pro-gastrin-releasing peptidpro-GRP (pro-GRP), and disease stage are recommended as tumor markers and risk factors for early prediction of adenocarcinoma transformation to SCLC (7, 22–24).

Tissue biopsy and NGS during disease progression are particularly important for the diagnosis of patients with EGFR mutations. The time of the transformation from NSCLC to SCLC usually takes more than 1 year (3, 25). The type of EGFR mutation before and after transformation is mostly consistent, and the biological behavior after transformation is similar to that of classical SCLC. However, most patients progress rapidly, with fewer effective treatments, shorter remission periods, and worse prognoses. In our case, the median survival time after transformation was only two months, while the primary SCLC had a median survival time of 8-13 months (26).

At present, the benefit of using TKIs after transformation is still unclear (27). Some studies have shown that patients with EGFR mutations had a clinical effective rate of 54% to platinum-etoposide after SCLC transformation, and the median estimated PFS was 3.4 months, but there was no response to checkpoint inhibitors, and the longest progression time was only 9 weeks (8); At the same time, some studies have shown that for the NSCLC patients with RET rearrangement, the effect of RET TKI treatment after transformation to SCLC may be poor (27, 28). Since there is no prospective randomized controlled study on the treatment regimes after lung adenocarcinoma transformation to SCLC at home and abroad, the standard chemotherapy regimes are generally selected as the main treatment for SCLC (29), but whether this regime is optimal needs further research.

There are also some other different opinions on the treatment of SCLC transformation. The 2020 Chinese Society of Clinical Oncology (CSCO) Guidelines for the Diagnosis and Treatment of Small Cell Lung Cancer recommend standard SCLC chemotherapy plus continued original EGFR-TKI as the treatment regimen for patients with transformed SCLC (Class III evidence, Class III recommendation) (7). In our case, it is advocated to pay attention to the combined treatment of adenocarcinoma and SCLC, as the patients with transformation may have more than one active clone at the same time. We found adenocarcinoma cells in the pericardial effusion, indicating that NSCLC still occurred which suggested the partial conversion.

This case provide clinical case evidence for EGFR-TKI combined with EP regimen when SCLC transformation occur. Related studies have also shown that combined cytotoxic chemotherapy and EGFR-TKI treatment can help improve the remission rate and PFS rate of SCLC transformation (30–32), so in addition to the treatment of SCLC, we should also pay attention to the treatment of the primary lung cancer type (6). Combining immunotherapy with cytotoxic chemotherapy as a strategy for beyond progression or maintenance therapy after the completion of cytotoxic chemotherapy may have greater clinical benefit than chemotherapy alone.

## Data Availability

The original contributions presented in the study are included in the article/supplementary material. Further inquiries can be directed to the corresponding author.
